# Genomic and transcriptomic analysis of genes involved in exopolysaccharide biosynthesis by *Streptococcus thermophilus* IMAU20561 grown on different sources of nitrogen

**DOI:** 10.3389/fmicb.2023.1328824

**Published:** 2024-01-29

**Authors:** Yuenan Wang, Qingting Peng, Yang Liu, Na Wu, Yanyan He, Xinrui Cui, Tong Dan

**Affiliations:** ^1^Key Laboratory of Dairy Biotechnology and Engineering, Ministry of Education, Inner Mongolia Agricultural University, Hohhot, China; ^2^Key Laboratory of Dairy Products Processing, Ministry of Agriculture and Rural Affairs, Inner Mongolia Agricultural University, Hohhot, China; ^3^Inner Mongolia Key Laboratory of Dairy Biotechnology and Engineering, Inner Mongolia Agricultural University, Hohhot, China

**Keywords:** *Streptococcus thermophilus*, different nitrogen media, exopolysaccharide biosynthesis, genome analysis, transcriptomic analysis

## Abstract

Exopolysaccharides (EPSs), which are produced by lactic acid bacteria, have been found to improve the texture and functionality of fermented dairy products. In a previous study, four nitrogen sources were identified as affecting the yield, molecular weight and structure of EPSs produced by *Streptococcus thermophilus* IMAU20561 in M17 medium. In this genomic and transcriptomics study, a novel *eps* gene cluster responsible for assembly of repeating units of EPS is reported. This *eps* cluster (22.3 kb), consisting of 24 open reading frames, is located in the chromosomal DNA. To explore the biosynthetic mechanisms in EPS, we completed RNA-seq analysis of *S. thermophilus* IMAU20561 grown in four different nitrogen sources for 5 h (log phase) or 10 h (stationary phase). GO functional annotation showed that there was a significant enrichment of differentially expressed genes (DEGs) involved in: amino acid biosynthesis and metabolism; ribonucleotide biosynthesis and metabolism; IMP biosynthesis and metabolism; and phosphorus metabolism. KEGG functional annotation also indicated enrichment of DEGs involved in amino acid biosynthesis, glycolysis, phosphotransferase system, fructose, and mannose metabolism. Our findings provide a better understanding the genetic traits of *S. thermophilus*, the biosynthetic pathways needed for the production of EPS, and a theoretical basis for screening dairy starter cultures.

## 1 Introduction

Many lactic acid bacteria (LAB) are widely used in medicine, dairy products, and biotechnology due to their generally recognized as safe (GRAS) status (Plavec and Berlec, 2020). Exopolysaccharides (EPS) are one of the most important secondary metabolites produced by LAB during metabolism; they are secreted externally to the cell surface and contribute to growth promotion and retardation of water loss from microbial cells (Angelin and Kavitha, 2020). As organic macromolecules, EPSs have complex and diverse structures and are widely employed in the fermented dairy products industry as thickeners, stabilizers, emulsifiers, and gelling agents (Daba et al., 2021; Tiwari et al., 2021). LAB EPSs also have beneficial effects on human health because of their antioxidant and antitumor properties and cholesterol-lowering abilities and also known for enhancing immunity and improving the gastrointestinal environment (Maeda et al., 2004; Laiño et al., 2016; Chen et al., 2022).

Several factors influence the yield and phenotypic characteristics of EPSs, such as carbon sources, nitrogen sources and incubation conditions (e.g., temperature, pH, agitation, oxygen levels and aeration) (Abd-Alla et al., 2018; Wu and Shah, 2018; Cheng et al., 2019). Recently researchers have focused their attention on EPS production by LAB on different carbon sources as essential components of the growth media (Oleksy-Sobczak and Klewicka, 2020). For example, the chemical composition, structure, morphology, and physicochemical properties of EPSs produced by *Lactobacillus rhamnosus* varied depending on the carbon source (Polak-Berecka et al., 2015). Yang et al. (2010) also reported that *L. rhamnosus* JAAS8 was capable of producing two forms of EPS, capsular and slime-polysaccharides, when grown in MRS broth or semi-defined medium with glucose as a carbon source. Similarly, nitrogen sources are important components in media and act as essential elements for growth (Karadeniz et al., 2021). Zhang et al. (2011) reported that growth and polymerization exopolysaccharides by *Streptococcus thermophilus* ST1 can be promoted by adding the protein concentrate to skimmed milk medium. However, the biosynthetic mechanisms responsible for exopolysaccharide production under different nitrogen sources are not clear. Hence, in this study, we used detailed multi-omics techniques to investigate EPS production mechanisms in *S. thermophilus* IMAU20561 (*S. thermophilus* IMAU20561) when grown in the presence of different nitrogen sources that influence the production of EPS.

In a previous study, we showed that the type of nitrogen source significantly affected yield, chemical composition and molecular weight of EPSs produced by *S. thermophilus* IMAU20561 (Liu et al., 2022). When soybean peptone was employed as the single nitrogen source, the amount of exopolysaccharide produced by this strain was 480.7 mg/L. When casein peptone was used as the single nitrogen source, the EPS produced had the largest molecular weight. There were significant differences in the structure of EPS when grown using different nitrogen sources. The EPS obtained on soybean medium mainly consisted of glucuronic acid, glucose, and galactose. The EPS obtained on micro tryptone medium was mainly made up of glucuronic acid, glucose, and galactose. The EPS obtained on casein peptone medium and basic medium M17 mainly comprised mannose, glucose, and galactose. This motivated us to study intracellular changes in relation to biosynthesis of bacterial EPSs. The phenotypic characteristics of EPS are complicated and regulated by genes associated with EPS biosynthesis, which are poorly understood in *S. thermophilus* IMAU20561. Therefore, in this study, we completed a genomic and transcriptomic analysis of *S. thermophilus* IMAU20561 grown in M17 medium using different nitrogen sources (soy peptone, tryptone, casein peptone) to examine the key regulatory genes involved in the EPS biosynthesis pathway and resulting EPS phenotypic characteristics in the *S. thermophilus* IMAU20561.

Based on the results for *S. thermophilus* ASCC 1275 (Padmanabhan et al., 2018) and preliminary observations of large differences in EPS production in the presence of different nitrogen sources, two time points (5, 10 h) were chosen for studying transcriptomics. The primary goal of this study was to investigate nitrogen source-associated changes in mRNA expression levels of *S. thermophilus* IMAU20561 to understand the regulatory mechanism driving the phenotypic characteristics of EPS. This research lays a new theoretical and practical foundation for further studies on the regulation of phenotypic characteristics of bacterial EPS.

## 2 Materials and methods

### 2.1 Bacterial strains and culture conditions

*Streptococcus thermophilus* IMAU20561 originated from yogurt sampled in the Zavkhan Province, Mongolia, and was used throughout this study (Liu et al., 2022). The *S. thermophilus* isolate was cultured at 37°C for 24 h in M17 liquid broth and then incubated under the same conditions in 50 and 500 ml M17 medium, at a 2% inoculation rate. Cells were collected as a pellet following centrifugation for 5 min at 4,000 *g* (4°C), washed twice with phosphate-buffered saline (PBS) at pH 7.4 containing 0.8% NaCl, 0.02% KCl, 0.02% KH_2_PO_4_, 0.115% Na_2_HPO_4_ and then suspended in PBS buffer.

*Streptococcus thermophilus* IMAU20561 was activated and then incubated at a 2% inoculation rate in either M17 medium, which contains a complex nitrogen source (5 g/L of soy peptone, 2.5 g/L of casein peptone, 2.5 g/L of peptone, 2.5 g/L of beef peptone, 5 g/L of yeast extract powder), or in medium in which the complex nitrogen source was replaced with either soy peptone (17.5 g/L), tryptone (17.5 g/L) or casein peptone (17.5 g/L) as sole nitrogen sources. After 24 h of incubation at 37°C, OD_600_ values were then recorded every hour and the growth curve of *S. thermophilus* IMAU20561 plotted for each medium.

### 2.2 DNA extraction

The Wizard^®^ Genomic DNA Purification Kit (Promega) was used to extract DNA from cells according to the manufacturer's instructions (Smith et al., 2003). Then, the integrity and quality of the extracted DNA fragments were confirmed by 1% agarose gel electrophoresis.

### 2.3 Gene prediction and functional annotation

The *S. thermophilus* IMAU20561 genome was sequenced using Glimmer v3.02 software (http://www.cbcb.umd.edu/software/glimmer/) (Delcher et al., 2007). Amino acid sequences were extracted from the annotated coding sequences and used for assignment and searched against Rapid Annotation using Subsystem Technology (RAST) (Brettin et al., 2015). Clusters of Orthologous Groups of proteins (COG) annotations were made to explore unigenes (http://www.ncbi.nlm.nih.gov/COG/) (Tatusov et al., 2003; Hyatt et al., 2010). Next, functional assignments were described by Gene Ontology (GO; http://www.geneontology.org) using Blast2GO software (Qi et al., 2016). Pathway assignments were mapped according to the Kyoto encyclopedia of genes and genomes (KEGG) database (http://www.genome.jp/kegg) (Li et al., 2021). A circular graphical map of *S. thermophilus* IMAU20561 was drawn using CGView (http://stothard.afns.ualberta.ca/cgview_server/) (Stothard et al., 2019) software.

### 2.4 Quantitative real-time PCR

Total RNA was extracted from *S. thermophilus* IMAU20561 grown on M17 medium containing different nitrogen sources using TRIzol reagent (Invitrogen) following the manufacturer's instructions. RNA extracts were treated with DNase I (Ambion) as recommended by the manufacturer and were measured at an absorbance of 260 nm using ND-2000 (NanoDrop Technologies). The purity of RNA was checked with an Agilent 2100 bioanalyzer (Agilent Technologies, Santa Clara, CA, United States). For cDNA synthesis, First Strand Master Mix and Super Script II reverse transcriptase (Invitrogen) were employed according to the manufacturer's instructions. The mixture was incubated at 50°C for 15 min followed by inactivation at 80°C for 2 min. The primers were designed by primer premier 5.0 software (Table 1) designed based on *S. thermophilus* IMAU20561 *eps* cluster sequences and other known genes involved in EPS biosynthesis. Quantitative real-time PCR was performed using the LineGene 9600 Plus RT-PCR detection system (Hangzhou Bori Technology Co., Ltd., China) and SYBR green PCR master mix (Applied Biosystems) as recommended by the manufacturer. Cycle conditions were 94°C for 2 min, followed by 40 cycles of denaturation at 94°C for 30 s, annealing at 60°C for 60 s, extension at 68°C for 120 s, and a final extension at 68°C for 7 min. The 16S rDNA gene was used as a reference gene for expression analysis, and the comparative critical threshold method (2^−Δ*ΔCt*^) method was used to calculate the relative expression of each target gene.

**Table 1:** Primers used in qRT-PCR.

**Primer name**	**Sequence (5^′^-3^′^)**	**Sequence (3^′^-5^′^)**
16S rDNA	GGTCTGTAACTGACGCTGAGG	GCACTGAAGGGCGGAAAC
*gene*0313	CCATATTGAACCAGAAACAG	ACCGATTTGATAAGCAGAAC
*gene*0359	AGATGAACAGTTGGATAAGG	TATCACAAATAAGACCAGCG
*gene*0526	GGATTCCCTATACAACAGAC	TACTCAGATGGCGTAATCAC
*gene*0861	TTGCCATTGACTACTACAAG	CTGCTTTTTCAAGAAGTGGG
*gene*0921	AAGCATTGTCCTTTTAGGTG	AGGAAAGTTGCAATTAGAGC
*gene*1059	TCGGGTGATTTCACTATCTG	AATCAAGCTAACCAAAAGGG
*gene*1252	CTTCAAACTTGATGCCAAAC	AAACCATGTTCAGTCAAACG
*gene*1289	GAACGCTTGGAAAAGATTAC	CACTTGAGCTGAAGACAATG
*gene*1345	GTTTTAGGGATTTCAGGAGG	ATCGTAAGACTTACATCTGG
*gene*1691	TGTTACTTTCATGCCAAGTG	TAGCATTGGCAAGTTAAGTC
*gene*1747	AAGAATTTTCTGAAGGGGAG	GGTATTCACATATTGTGGAG
*gene*1871	TAGGTGACGCTCATATTTTG	TTGATCAGATACGTCAACGC
*gene*1065	GGCAATCTTAGTTTTAGGTG	GTATTCTACGAGGGGATTTA
*gene*1452	GACGATTCTTCAGAATCTGC	TCAGATTTCAAGATGTCAGC

### 2.5 Transcriptional analysis

Transcriptional analysis was performed on genes involved in EPS biosynthesis in *S. thermophilus* IMAU20561 grown for 5 h and 10 h in M17 medium containing different nitrogen sources, such as either soy peptone, tryptone, casein peptone as the sole sources or the complex nitrogen source typical of the M17 medium. The construction of the transcriptome library was done by the Shanghai Meiji Biological Analysis and Testing Co., Ltd. (Meiji, Shanghai) with the TruSeqTM RNA sample preparation Kit (Illumina, San Diego, CA). The mRNA was fragmented using metal ions and double-stranded cDNA was reverse transcribed with random primers using the SuperScript double-stranded cDNA kit (Invitrogen, CA). The second cDNA strand was synthesized, with dUTP instead of deoxythymidine triphosphate (dTTP), cDNA ends were patched with End Repair Mix and phosphorylated at the 5′ end and adenylated at the 3′ end. The cDNA library-enriched and the PCR were amplified using Phusion DNA polymerase (NEB). RNA-seq sequencing was done using Illumina HiSeq X Ten (2 × 150 bp).

### 2.6 Data processing and analysis

The raw image signal obtained by high-throughput sequencing (Illumina HiSeq X Ten) was transformed into sequenced reads in the FASTQ format and filtered to obtain clean reads by removing adapter sequences, low-quality sequences (QV <Q20), sequences with more than 10% N. Genomic localization analysis of filtered sequences of *S. thermophilus* IMAU20561 (GenBank accession: GCA_021294245.1) was performed using Bowtie2 (http://bowtie-bio.sourceforge.net/bowtie2/index.Shtml) (Nie et al., 2021). Moreover, the expected number of Fragments Per Kilobase of transcript sequence per Million base pairs sequenced (FPKM) value was used to represent the expression level. Functional pathway enrichment analysis was performed on the KEGG pathway including analysis of the metabolic network. GO analysis of DEGs were done using Goatools (https://github.com/tanghaibao/GOatools). GO terms with the corrected *p*-values <0.05 were considered as significantly enriched in DEGs.

The results were expressed as the mean ± SD of three replicates. All digital analyses were carried out using the SPSS (IBM, USA). A *p* value <0.05 was deemed statistically significant.

## 3 Results

### 3.1 General characteristics of the *S. thermophilus* IMAU20561 genome

**Figure 1:**
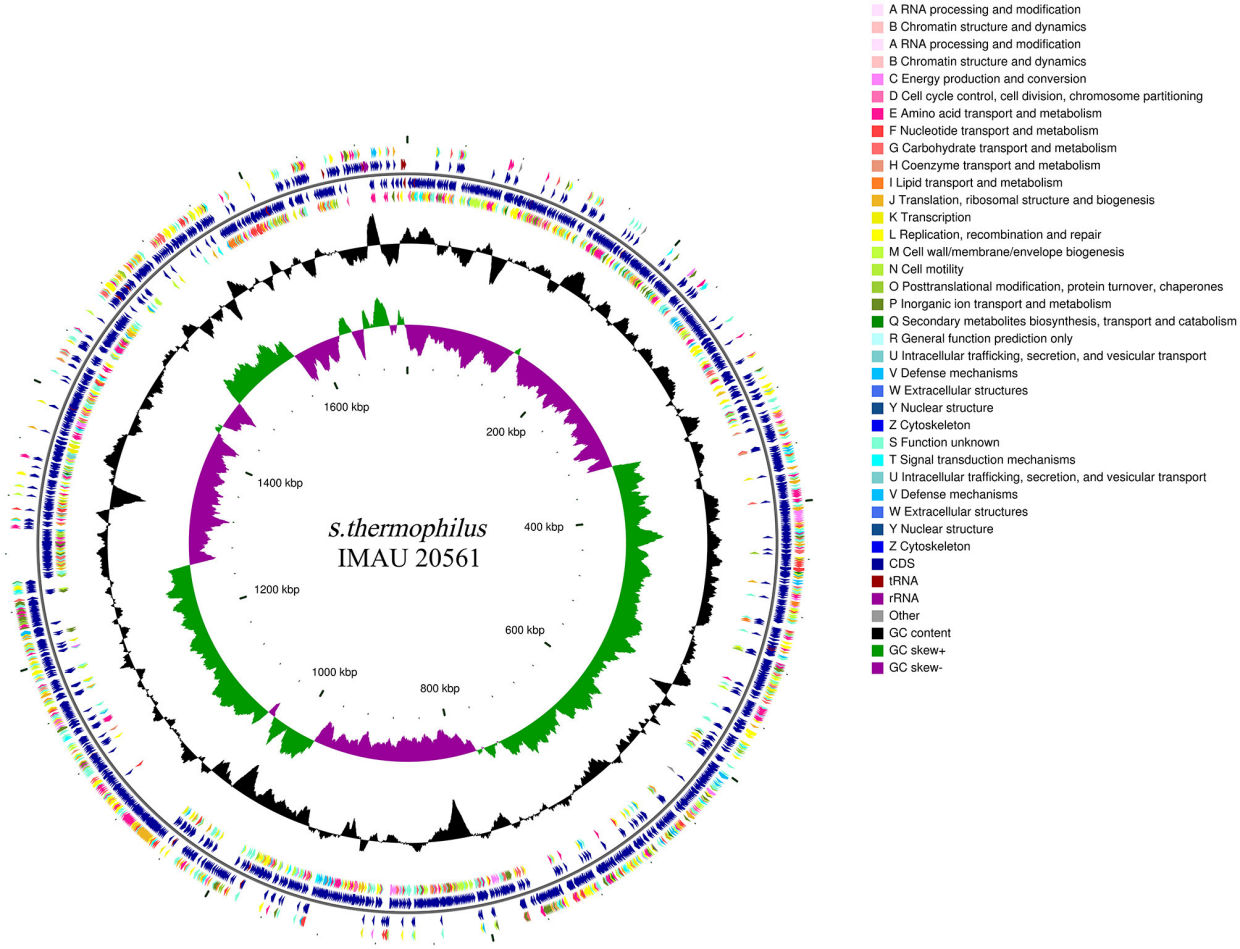
A circular graphical map of the genome of *Streptococcus thermophilus* IMAU20561.

The complete genome sequence of *S. thermophilus* IMAU20561 contains a circular 1,716,258 bp chromosome with 39.03% GC content, with N50 and N90 values of 127,615 bp and 31,779 bp, respectively; no plasmids were identified (Figure 1). The total length of 1,914 CDS coding genes, four rRNA operons and 42 tRNAs was 1,436,388 bp, accounting for 83.69% of the total genome (DDBJ accession no. GCA_021294245.1). From outer to inner rings, the first and fourth circles were on the forward strand, the second and third circles represented the reverse of CDS, tRNA, rRNA, the fifth circle represented GC content and the sixth circle represented GC-Skew. Using the original genome of the model strain *S. thermophilus* NCTC 12958 as reference, the average nucleotide (ANI) value of the two strains was calculated. The results showed that the average ANI value of the two strains was 98.42%. The strains showed a high similarity with the reference genome, indicating that *S. thermophilus* IMAU20561 belonged to the same species as the reference strain.

### 3.2 Functional annotation

**Figure 2:**
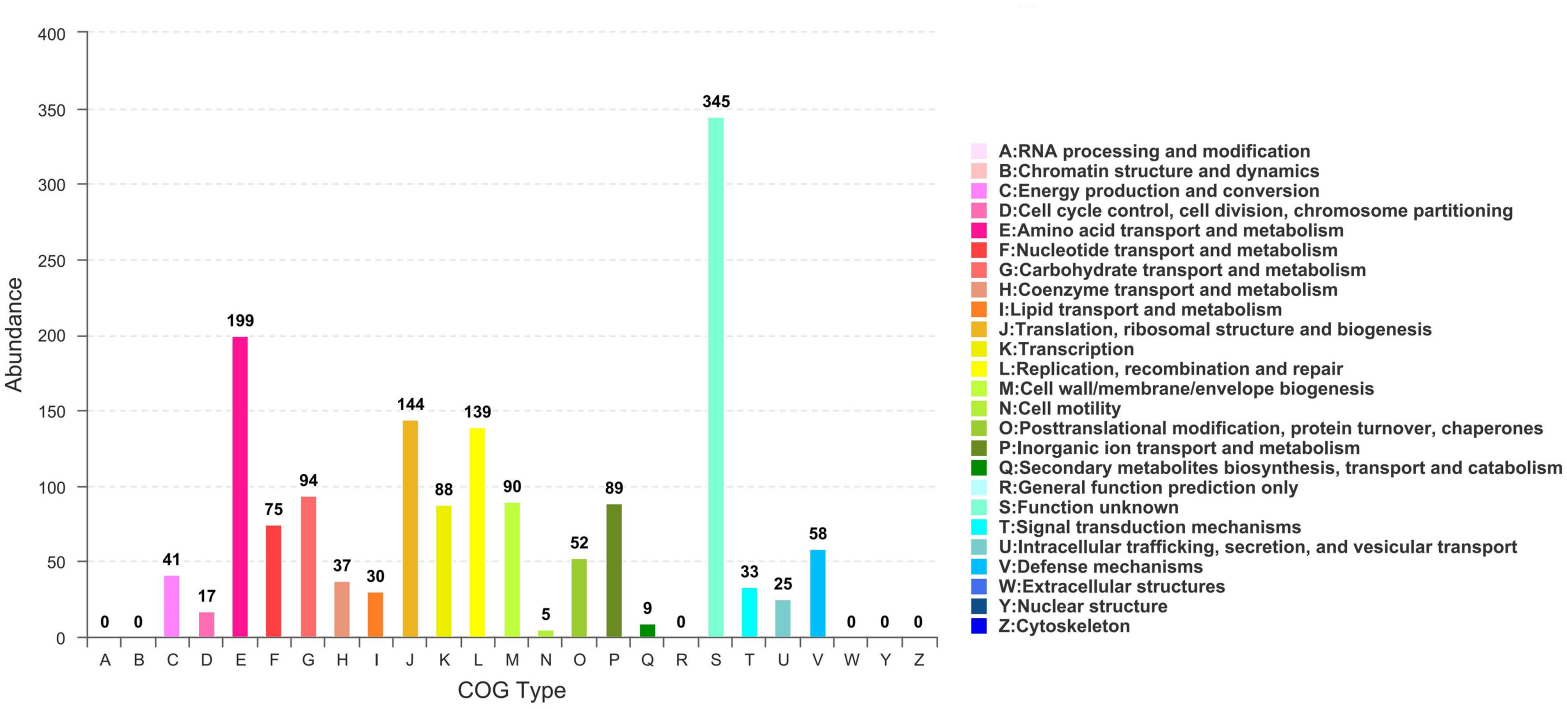
COG functional classification of *Streptococcus thermophilus* IMAU20561. The *x*-axis is the COG type. The *y*-axis shows the number of unigenes.

**Figure 3:**
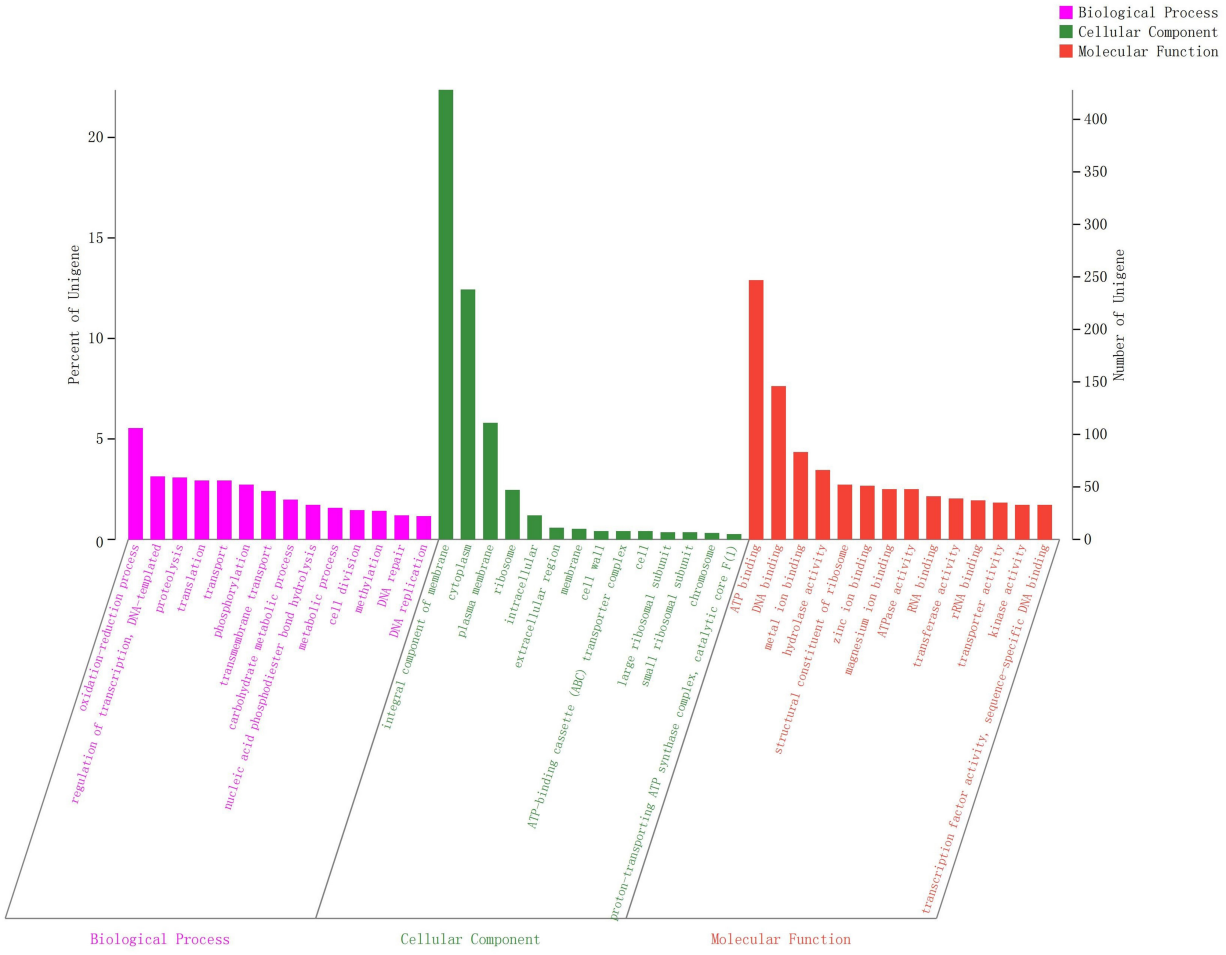
GO functional classification of *Streptococcus thermophilus* IMAU20561. The *x*-axis indicates the three main ontologies including biological processes, cellular components, and molecular function. The *y*-axis represents the number of genes in each category.

**Figure 4:**
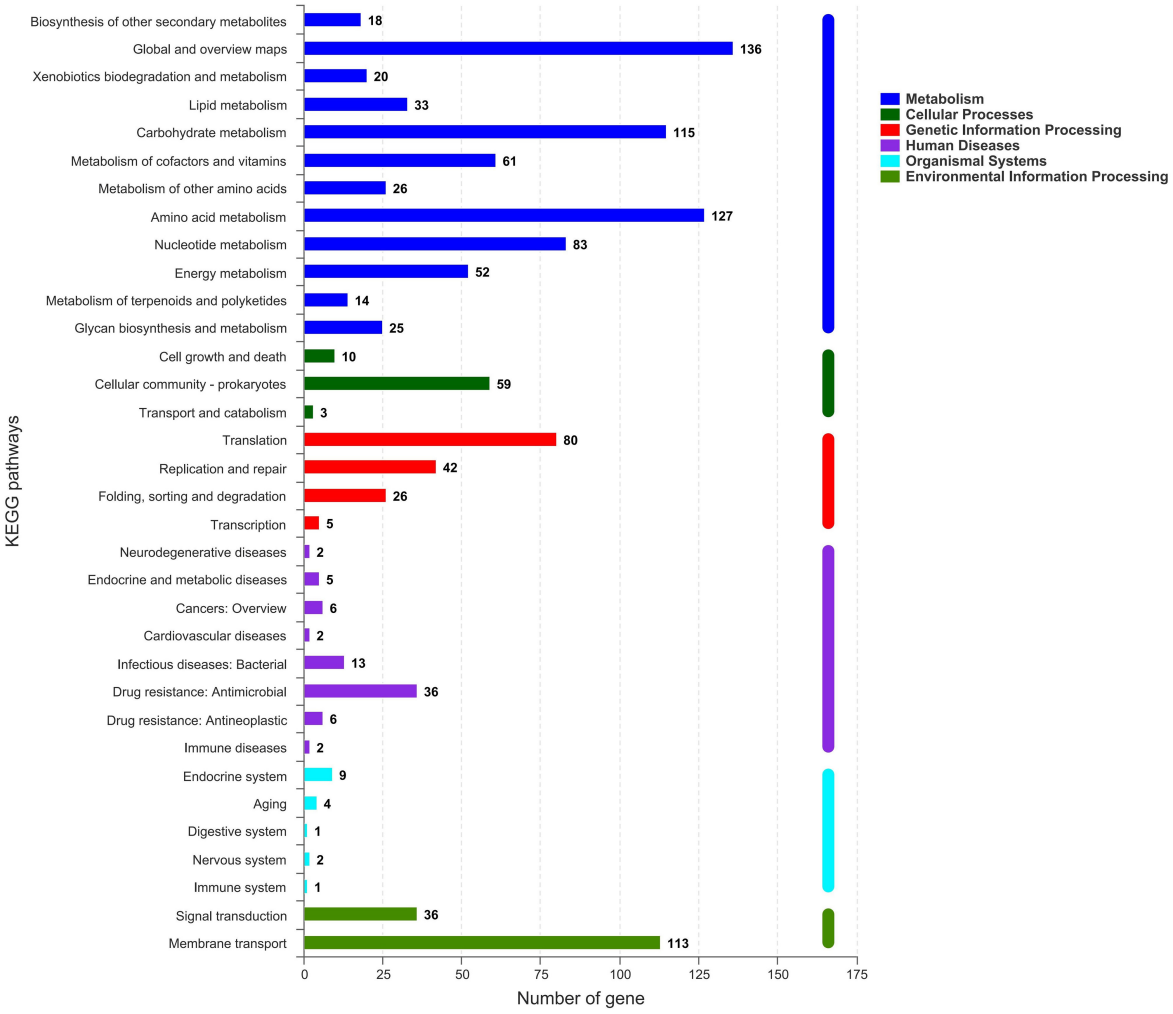
KEGG metabolic pathways of *Streptococcus thermophilus* IMAU20561. The *x*-axis indicates the number of genes. The *y*-axis indicates the KEGG pathways.

All generated unigenes were aligned against COG, GO, and KEGG databases and annotated with function (Figures 2–4). The COG annotation (Figure 2) indicated that a total of 1,549 genes were obtained from the predicted coding region of the genome; among the identified genes, 345 were of unknown function, 199 were involved in amino acid transport and metabolism, 144 were associated with ribosome structure, translation and biosynthesis, 139 appeared to be involved in replication, recombination and repair, 99 were involved in carbohydrate transport and metabolism, and 365 genes with potential biological functions were not annotated. For the COG annotations, 1,414 genes in the genome were annotated using the GO database and assigned to three major functional classifications (Figure 3), including “biological processes,” “cellular components,” and “molecular function.” A total of 1,108 genes were obtained by KEGG annotation, which included 127 genes for amino acid metabolism (11.5%) and 114 for regulation of the carbohydrate metabolism (10.4%; Figure 4). In addition, some genes associated with membrane transport, nucleotide metabolism and translation were also enriched.

### 3.3 Identification of the EPS biosynthetic gene cluster

**Figure 5:**
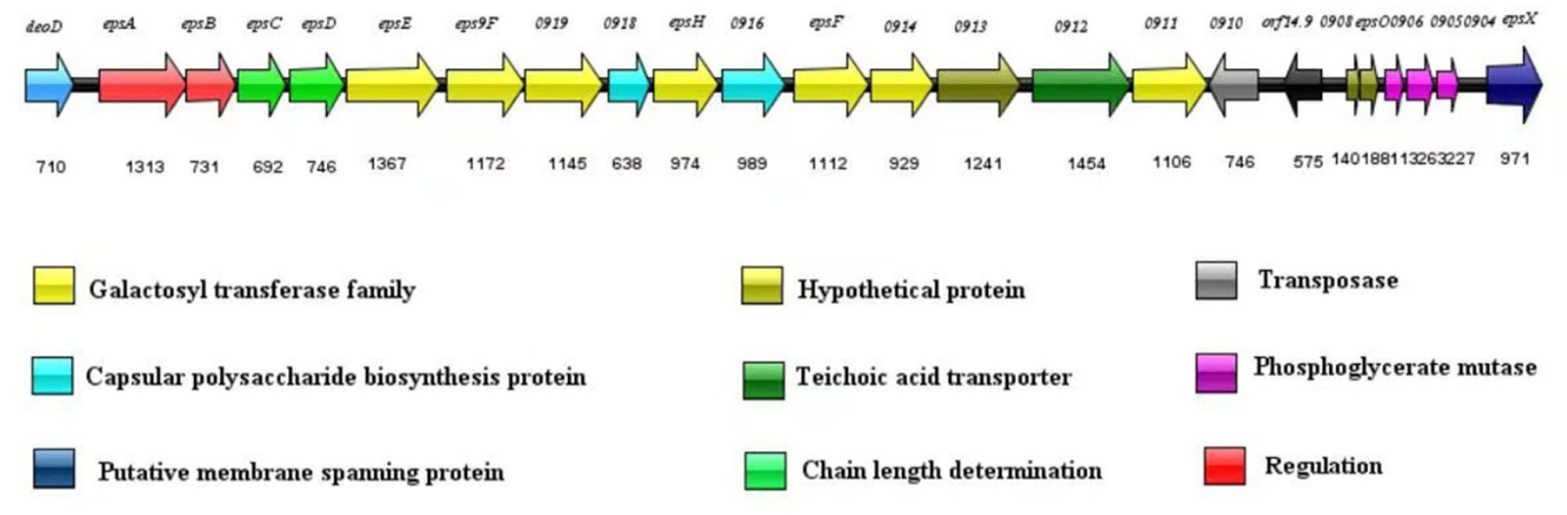
Exopolysaccharide gene cluster in *Streptococcus thermophilus* IMAU20561.

The nucleotide sequence of the *eps* gene cluster in *S. thermophilus* IMAU20561 (22.3 kb) was determined by gene annotation. In the gene cluster, 24 ORFs were found by computational analysis (Figure 5). The gene *deo*D was located upstream of gene *eps*A, followed by *eps*B, *eps*C, *eps*D, *eps*E, *eps*9F, and *gene* 0919. Two capsule biosynthetic proteins (*gene* 0918, *gene* 0916) were present between *gene* 0919, *eps*H, and *eps*F. After *eps*F, *gene* 0914, *gene* 0913, *gene* 0912, and *gene* 0911 were found in this *eps* cluster. A transposase-like gene *(gene* 0910) and *orf* 14.9 were found to inserted in the cluster in the opposite orientation. Downstream of *orf* 14.9, two hypothetical proteins, three phosphoglycerate mutases and a putative membrane spanning protein were found.

The predicted functions of *eps* genes were divided into four regions covering regulation, determining chain length, biosynthesis of the repeating unit, polymerization, and export. Upstream of the *eps* gene cluster was *deo*D, encoding purine nucleotide phosphorylase, involved in the biosynthesis and catabolism of nucleotides, and similar to a purine nucleoside phosphatase in *S. thermophilus* LMD-9 (ID: Q03K54.1), with 100% sequence identity (Goh et al., 2011). As a transcriptional regulator of the LytR family, *eps*A had 97.81% homology with *eps*A (ID: ADQ63266.1) identified from *S. thermophilus* ND03 (Sun et al., 2011); *eps*B also showed a significant identity with *eps*B (97.62%) from *S. thermophilus* MN-ZLW-002 (ID: AFJ83638.1) (Luo et al., 2022). The gene *eps*C displayed a 99% identity with *eps*C in *S. thermophilus* M17PTZA496 (ID: ETW89010.1) and *eps*D showed a 99.86% identity with the *eps*D in *S. thermophilus* MTCC-5461 (ID: ELW74268.1) (Prajapati et al., 2013; Da Silva Duarte et al., 2018). The genes *eps*E, *eps*9F, *eps*H and *eps*F encoding glycosyltransferases are responsible for synthesis of EPS repeating units. Among These *eps*E, those encoding a priming glycosyltransferase showed a 100% homology with the glycosyltransferase gene of *S. thermophilus* TH1436 (ID: 1423145), which is responsible for transferring sugar nucleotides to the isoprenoid glycolipid carrier and considered the first step in synthesis of the repeat unit (Giaretta et al., 2018). The gene *eps*9F, encoding a glycosyltransferase, showed a 99.21% homology with the glycosyltransferase gene in *S. thermophilus* TH1435 (D: ETE41202.1) (Treu et al., 2014). To build the repeating units, *eps*F and *eps*H transferred sugar nucleotides to the *eps*E one after another. *eps*F and *eps*H showed a high similarity (99.73%, 99.79%) to the glycosyltransferase in *S. thermophilus* ND03 (ID: ADQ63266.1). In addition, three genes encoding glycosyltransferases were found, namely *gene* 0919, *gene* 0914, and *gene* 0911. *Gene* 0916 and *gene* 0918 are responsible for encoding capsular polysaccharide biosynthesis protein and respectively had a 98.83 and 99.74% sequence identity with the capsular synthesis protein gene in *S. thermophilus* ASCC 1275 (ID: AIC24645.1) and *S. thermophilus* MN-ZLW-002 (ID: AFJ83638.1) (Padmanabhan et al., 2020; Luo et al., 2022). Downstream of the *eps* gene cluster was *orf* 14.9 with a direction opposite to the *eps* gene cluster, which is associated with cell growth. The genes *eps*O and *eps*X, located downstream of *orf* 14.9, are involved in the transfer and export of EPS and showed a 99.62 and 100% sequence identity to the *S. thermophilus* M17PTZA496 (ID: ETW89010.1) and *S. thermophilus* LMD-9 (ID: Q03K54.1), respectively (Goh et al., 2011; Da Silva Duarte et al., 2018). *Gene* 0906 had a 99.77% homology to the phosphatase gene in *S. thermophilus* LMG 18311 (ID: AAV60888.1) (Blomqvist et al., 2010). *Gene* 0910 had a 100% similarity to a putative transposase in *S. thermophilus* MTCC-5461 (ID: ELW74268.1) (Prajapati et al., 2013). *Gene* 0904 and *gene* 0912 respectively had a 100% and 99.53% similarity to the putative phosphoglycerate mutase and teichoic acid transporter in *S. thermophilus* LMD-9 (ID: Q03K54.1) (Goh et al., 2011).

### 3.4 Transcriptional analysis

**Figure 6:**
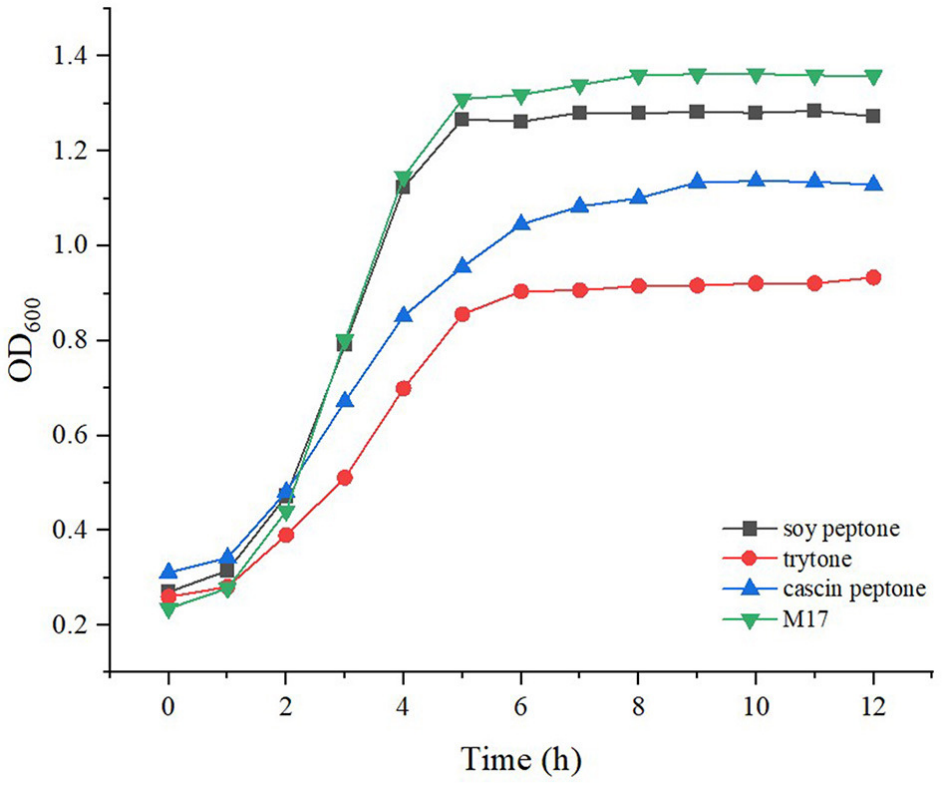
Growth curves of *Streptococcus thermophilus* IMAU20561 grown under different nitrogen sources.

*Streptococcus thermophilus* IMAU20561 initially grew slowly and then its growth began to accelerate after 2 h of culture (Figure 6). After 6 h, the cells entered the stationary phase (Figure 6). Therefore, the end of the exponential phase at 5 h and the stationary phase at 10 h were chosen for transcriptional analysis.

**Figure 7:**
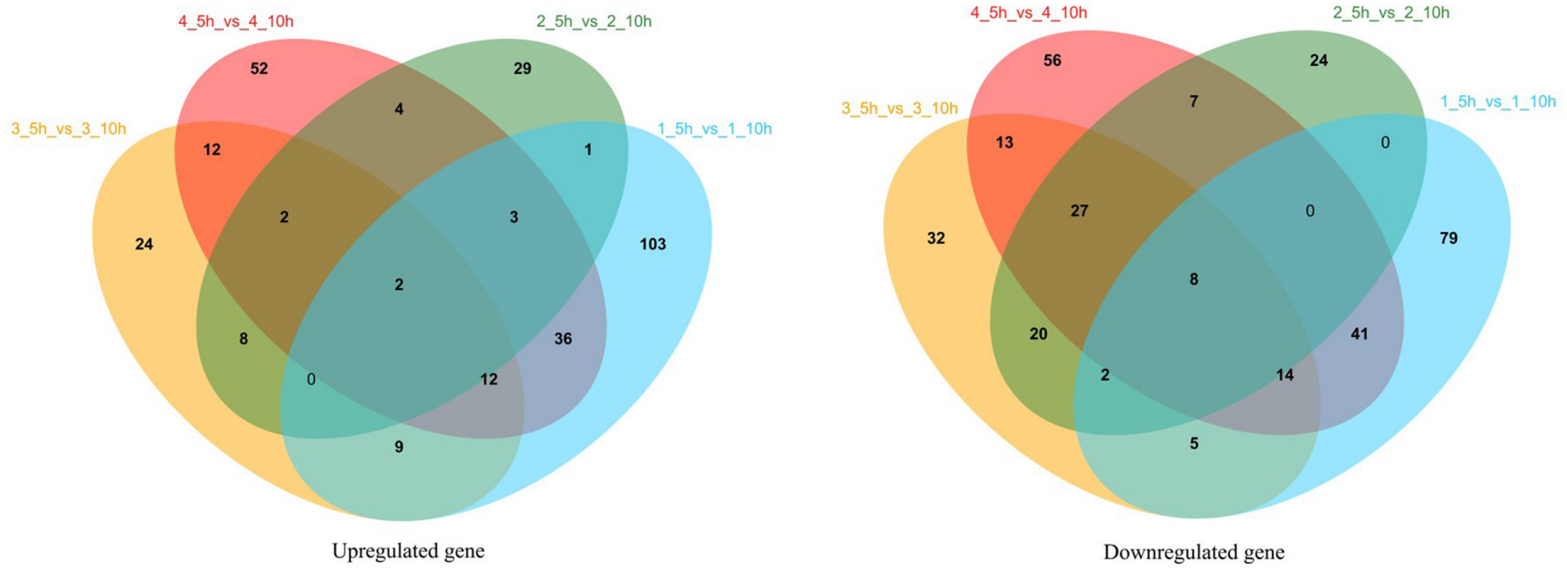
Venn analysis of gene expression during growth in M17 media with different nitrogen sources at logarithmic growth phase (5 h) and stationary growth phase (10 h). 1_5 h_vs_1_10 h represents the 5 and 10 h gene expression analysis results of IMAU20561 in soy protein medium; 2_5 h_vs_2_10 h represents the 5 and 10 h gene expression analysis results of IMAU20561 in tryptone medium; 3_5 h_vs_3_10 h represents the 5 and 10 h gene expression analysis results of IMAU20561 in casein peptone medium; 4_5 h_vs_4_10 h represents the 5 and 10 h gene expression analysis results of IMAU20561 in M17 medium.

Transcriptome profiles enabled the investigation of the variation in gene expression in the presence of different nitrogen sources in *S. thermophilus* IMAU20561. The cDNA library construction and sequencing of *S. thermophilus* MAU20561 generated 26,698,687 to 32,902,934 reads. A minimum of 83.57% of the genome could be mapped to the reference genome of this species. Gene expression during the growth in M17 media supplemented with different nitrogen sources at the logarithmic growth phase (5 h) and stationary growth phase (10 h) are presented as a Venn diagram of genes (Figure 7). Volcano plots revealed clear distinctions in the differential gene expression between different nitrogen sources under the above conditions (Supplementary Figure S1). Among these genes, there were 715 significantly regulated transcripts; 352 genes were upregulated and 363 genes were downregulated in the presence of soy peptone. In the presence of tryptone, 198 genes were upregulated while 255 were downregulated. In the presence of casein peptone, there were 578 significantly regulated transcripts; 255 genes were upregulated while 323 genes were downregulated. In the presence of the complex medium (M17), 315 genes were upregulated and 375 genes were downregulated.

### 3.5 KEGG pathway enrichment analysis

The 16 groups of DEGs from the KEGG enrichment analysis are graphically displayed in a scatter plot (Supplementary Figure S2). The “rich factor” refers to the ratio of the number of DEGs enriched and the number of annotated background genes in the KEGG pathway and this factor is often used to represent the degree of KEGG enrichment. In Supplementary Figure S2, the highest rich factor, gene numbers and lower FDR value indicates greater enrichment. When soy peptone was the sole nitrogen source, the significantly enriched DEGs were mainly related to biosynthesis of histidine, phenylalanine, tyrosine, and tryptophan. When tryptone was the sole nitrogen source, the significantly enriched DEGs were mainly related to metabolic pathways of ribosomes, and some secondary metabolites such as the biosynthesis of streptomycin, neomycin, and gentamicin. When casein peptone was the sole nitrogen source, the significantly enriched DEGs were mainly in ribosome, histidine, and tyrosine biosynthesis. In the entire M17 medium with a complex nitrogen source, the significantly enriched DEGs were mainly related to histidine biosynthesis, peptidoglycan biosynthesis and the glycolysis/gluconeogenesis pathway. The most significantly involved pathways during nitrogen enrichment were the metabolism and biosynthesis pathway of nitrogen metabolism. Among them, histidine metabolism, tryptophan metabolism and phenylalanine metabolism are closely related to the biosynthesis of exopolysaccharides. Biosynthesis and metabolism of histidine involves the glycolytic pathway (EMP), the tricarboxylic acid pathway (TCA) and the pentose phosphate metabolism pathway (HMP). Anthranilic acid as a precursor for tryptophan synthesis in the carbohydrate metabolism can be beneficial to the production of tryptophan.

### 3.6 Genes involved in the biosynthesis of exopolysaccharides

We found that the exopolysaccharide yield of M17 was 150.8 and 480.7 mg/L when soy protein was the only nitrogen source, which was more than two times higher than that of M17. In addition, the EPS yield of trypsin medium was 175 mg/L, while the minimum EPS yield of casein peptone medium was only 28.1 mg/L (Liu et al., 2022). Therefore, we will continue to study exopolysaccharides under different culture conditions and analyze the expression of genes related to exopolysaccharide synthesis.

#### 3.6.1 Glycolysis or gluconeogenesis

**Figure 8:**
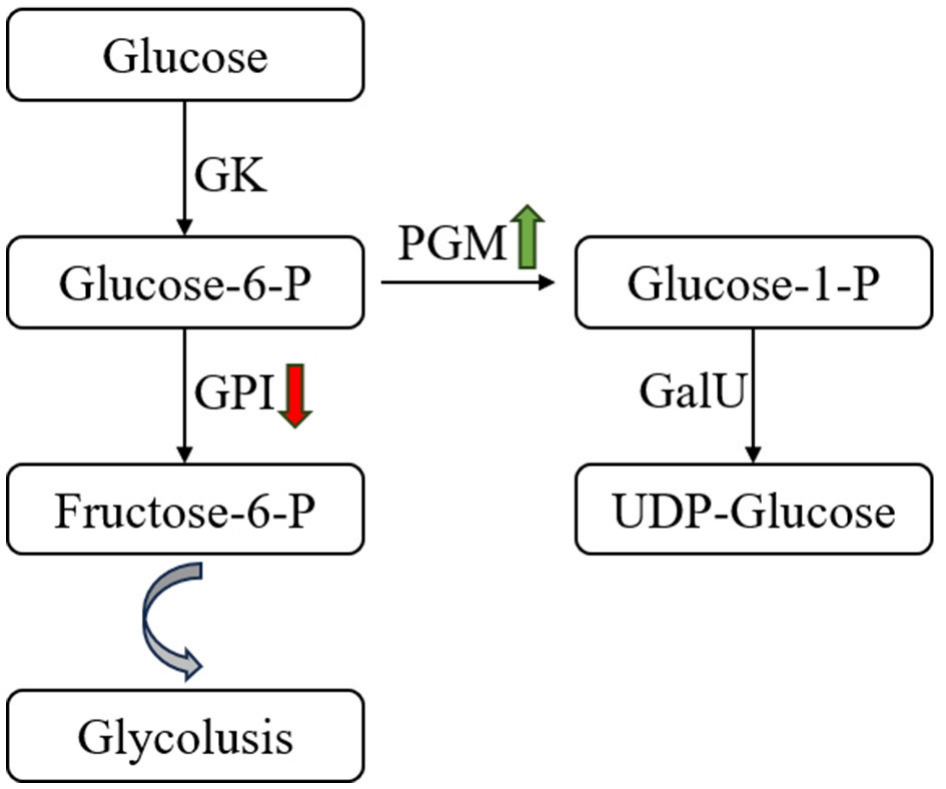
Changes in glucose metabolism pathway of *Streptococcus thermophilus* IMAU20561 during continuous culture. GK, glucokinase; GPI, glucose-6-phosphate isomerase; PGM, α-phosphoglucomutase; GalU, UDP-glucose pyrophosphorylase.

Glycolysis or gluconeogenesis is the main pathway of monosaccharide metabolism in LAB. In *S. thermophilus* MAU20561 culture at 5 and 10 h, a total of 32 genes involved in the glycolytic processes and gluconeogenesis were identified and 18 genes were significantly differentially expressed (Table 2). Of them, 15 genes were upregulated and three genes were downregulated when soy peptone was the sole nitrogen source; *gal*M, *pyk, pfk*A, and *bgl*A were upregulated by 1.8, 1, 1.2, and 1.4 times, respectively. Similar results were also found when tryptone was the sole nitrogen source where 15 genes were upregulated and three genes were downregulated; *adh*E was upregulated by 1.1 times. When casein peptone was the sole nitrogen source, 14 genes were upregulated and four genes were downregulated; *adh*E, *adh*E, and *adh*P were upregulated by 2.1, 1.8, and 1.1 times, respectively. When full M17 with a complex nitrogen source was used, 15 genes were upregulated and three were downregulated; *ldh* was upregulated by 1.5 times while *pgm, pyk*, and *glk* were upregulated by 0.82, 0.76, and 0.82 times, respectively. Phosphoglucomutase and glucose-6-phosphate isomerase were the key enzymes active in the glycolytic pathway. After glucose is converted to glucose-6-phosphate, glucose-1-phosphate and fructose-6-phosphate are generated under the catalytic action of phosphoglucomutase and glucose-6-phosphate isomerase, respectively (Cui et al., 2017). In this study, the phosphoglucomutase gene (*pgm*) was significantly upregulated while glucose-6-phosphate isomerase gene (*gpi*) was downregulated in the media with different nitrogen sources, indicating that, after 10 h of incubation, the production of precursor of UDP-glucose was found to have increased yields during the biosynthesis of precursor metabolites, glucose-1-phosphate, in *S. thermophilus* IMAU20561 (Figure 8). This result is consistent with the RT-qPCR gene expression analysis results (Section 3.7).

**Table 2:** Expression of gluconeogenic key genes in M17 medium with soy peptone, tryptone or casein peptone as the only nitrogen source.

**Gene ID**	**Gene names**	**1_10 h/1_5 h**		**2_10 h/2_5 h**		**3_10 h/3_5 h**		**4_10 h/4_5 h**	
		**Log**_2_ **FC**	***p*** **adjust**	**Log**_2_ **FC**	***p*** **adjust**	**Log**_2_ **FC**	***p*** **adjust**	**Log**_2_ **FC**	***p*** **adjust**
gene1879	*gapA*	0.624 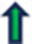	6.16E−23	0.446 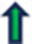	2.92E−17	−0.017 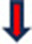	0.874081	0.544 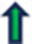	1.05E−11
gene1745	*galM*	1.817 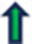	3.22E−150	0.445 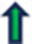	3.72E−08	0.291 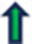	0.000452	1.183 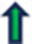	3.97E−41
gene1636	*pgm*	0.484 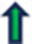	9.12E−17	0.420 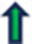	2.05E−14	0.568 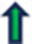	2.69E−17	0.821 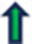	3.62E−30
gene1591	*fbaA*	0.308 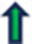	2.57E−08	0.495 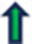	2.83E−17	0.126 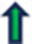	0.121282	0.959 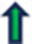	3.60E−28
gene1577	*adhE*	0.223 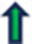	6.04E−01	1.100 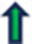	3.28E−09	2.116 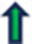	5.37E−17	0.664 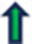	0.061251
gene1574	*adhE*	0.525 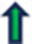	4.05E−01	1.127 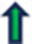	1.52E−06	1.817 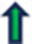	1.20E−17	1.090 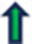	2.15E−05
gene1452	*malT*	0.701 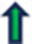	1.49E−01	0.250 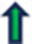	4.33E−03	0.555 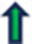	1.73E−09	0.333 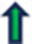	0.000300
gene1449	*gpi*	−0.210 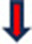	2.58E−14	−0.066 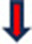	3.05E−01	−0.242 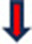	0.000137	−0.146 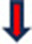	0.107396
gene1253	*pyk*	1.046 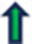	8.86E−04	0.153 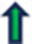	4.11E−03	0.261 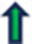	7.27E−05	0.762 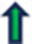	7.12E−35
gene1252	*pfkA*	1.177 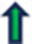	7.07E−76	−0.085 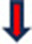	1.29E−01	0.037 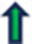	0.646081	0.398 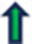	9.08E−09
gene1244	*gpmA*	0.995 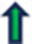	1.25E−75	0.83 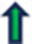	2.97E−38	0.234 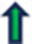	0.002158	0.995 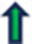	4.97E−47
gene0992	*ldh*	0.942 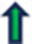	2.09E−58	0.344 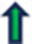	4.33E−08	−0.335 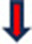	6.87E−06	1.523 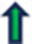	5.38E−93
gene0883	*lpd*	−1.941 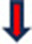	2.17E−160	−0.444 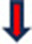	7.62E−12	−0.969 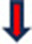	2.82E−45	−1.443 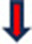	5.17E−54
gene0749	*adhP*	0.877 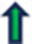	1.19E−09	0.377 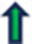	6.30E−03	1.089 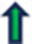	2.71E−23	1.014 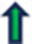	1.46E−10
gene0689	*glk*	0.617 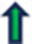	6.97E−22	0.605 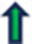	4.80E−17	0.289 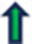	0.000118	0.820 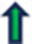	2.11E−29
gene0611	*ldh*	−1.001 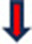	7.20E−34	0.294 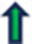	8.42E−06	−0.334 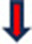	5.21E−07	−0.562 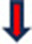	3.99E−09
gene0215	*bglA*	1.382 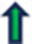	1.77E−16	0.615 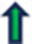	0.00774	0.582 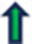	0.00662	1.051 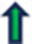	3.84E−07
gene0214	*bglA*	1.418 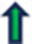	2.53E−14	0.243 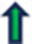	0.46933	0.115 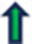	0.71400	0.505 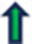	0.057281

1_10 h/1_5 h represents the 10 and 5 h gene expression analysis results of IMAU20561 in soy protein medium; 2_10h/2_5 h represents the 10 and 5 h gene expression analysis results of IMAU20561 in tryptone medium; 3_10 h/3_5 h represents the 10 and 5 h gene expression analysis results of IMAU20561 in casein peptone medium; 4_10 h/4_5 h represents the 10 and 5 h gene expression analysis results of IMAU20561 in M17 medium; Log_2_FC (stress/control): the logarithm value of the difference factor of this gene between two samples with the base of 2; p adjust: test result of the significant difference between the two samples of this gene. Green represents upregulation and red shows downregulation.

#### 3.6.2 Amino and nucleotide sugar metabolism

To become nucleotide sugars, monosaccharides must be activated by a high-energy donor, which enables them to be used in exopolysaccharide biosynthesis (Fan et al., 2015). Amino sugars that are present in exopolysaccharides are derived from sugar phosphate or sugar–nucleotide, and the corresponding amino sugar derivative is catalyzed by a specific aminotransferase or an amido transferase (Skarbek and Milewska, 2016). Some DEGs were involved in amino and nucleotide sugar metabolism in *S. thermophilus* MAU20561 under the influence of four selected nitrogen sources. Comparing the cultures at 5 and 10 h, 28 genes related to amino sugar and nucleotide sugar metabolism were identified. A total of 15 genes involved in amino and nucleotide sugar metabolism were significantly differentially expressed (Table 3). When soy peptone was the sole nitrogen source, 14 genes were upregulated and one gene was downregulated; *gal*E, *gal*T, *gal*K*, glm*U*, nag*A, and *man*A were upregulated by 1.12, 1.04, 0.97, 1.06, 1.06, and 0.93 times, respectively. Similar results were also found when tryptone was the sole nitrogen source in which case five genes were upregulated and three genes were downregulated genes; among them, *pgm* was slightly upregulated. When casein peptone was the sole nitrogen source, four genes were upregulated and three genes were downregulated; *gal*E and *nag*A were upregulated by 0.822 and 0.766 times, respectively. When the M17 medium with a complex nitrogen source was used for the culture, eight genes were significantly upregulated and four genes were downregulated; *gal*E, *gal*T, and *pgm* were upregulated by 0.878, 0.835, and 0.821 times, respectively.

**Table 3:** Expression of genes related to amino sugar and nucleotide sugar metabolism.

**Gene ID**	**Gene names**	**1_10 h/1_5 h**		**2_10 h/2_5 h**		**3_10 h/3_5 h**		**4_10 h/4_5 h**	
		**Log**_2_ **FC**	***p*** **adjust**	**Log**_2_ **FC**	***p*** **adjust**	**Log**_2_ **FC**	***p*** **adjust**	**Log**_2_ **FC**	***p*** **adjust**
gene1744	*galE*	1.124 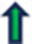	4.84E−42	0.281 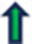	1.72E−07	0.325 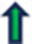	1.23E−07	0.878 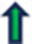	3.02E−45
gene1855	*galU*	0.424 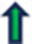	4.03E−10	0.234 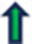	0.000673	0.393 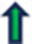	4.21E−08	0.310 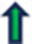	1.89E−06
gene1743	*galT*	1.041 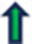	1.12E−45	0.521 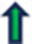	1.21E−15	0.572 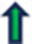	2.81E−17	0.835 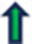	5.35E−23
gene1742	*galK*	0.973 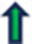	2.30E−45	0.403 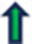	2.41E−08	0.531 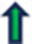	9.14E−19	0.758 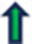	1.24E−13
gene1636	*pgm*	0.484 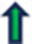	9.12E−17	0.420 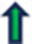	2.05E−14	0.568 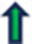	2.69E−17	0.821 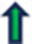	3.62E−30
gene1289	*galE*	0.180 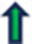	1.44E−02	0.836 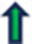	7.44E−34	0.822 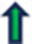	1.36E−27	0.684 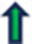	2.38E−13
gene1065	*galE*	0.740 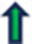	9.06E−03	−0.701 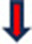	4.11E−03	−0.346 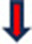	0.131769	−0.109 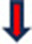	7.17E−01
gene1064	*galE*	0.760 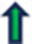	3.88E−02	−0.195 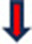	4.69E−01	0.351 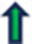	0.197485	−0.084 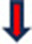	8.15E−01
gene0689	*glk*	0.617 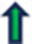	6.97E−22	0.605 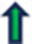	4.80E−17	0.289 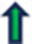	0.000117	0.820 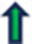	2.11E−29
gene0519	*glmU*	1.069 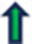	4.37E−72	0.089 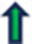	2.43E−01	0.053 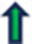	0.419866	0.558 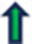	2.59E−18
gene0503	*nagB*	0.691 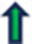	5.53E−05	−0.926 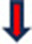	9.09E−10	0.410 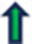	0.002119	1.281 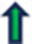	6.28E−12
gene0464	*nagA*	1.062 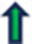	1.80E−20	0.768 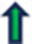	2.17E−18	0.767 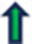	5.21E−16	0.989 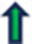	2.24E−15
gene0311	*scrK*	0.362 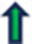	2.67E−07	0.484 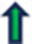	8.47E−11	0.324 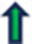	0.000123	0.229 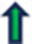	0.013362
gene0310	*manA*	0.937 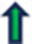	1.71E−08	0.637 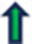	6.38E−08	0.654 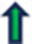	1.34E−08	0.468 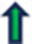	0.001367
gene0138	*murA*	−0.155 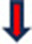	1.47E−01	−0.985 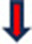	7.12E−47	−0.734 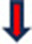	1.72E−28	−1.066 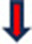	2.04E−19

1_10 h/1_5 h represents the 10 and 5 h gene expression analysis results of IMAU20561 in soy protein medium; 2_10h/2_5 h represents the 10 and 5 h gene expression analysis results of IMAU20561in tryptone medium; 3_10 h/3_5 h represents the 10 and 5 h gene expression analysis results of IMAU20561 in casein peptone medium; 4_10 h/4_5 h represents the 10 and 5 h gene expression analysis results of IMAU20561 in M17 medium; Log2FC (stress/control): the logarithm value of the difference factor of this gene between two samples with the base of 2; p adjust: test result of the significant difference between the two samples of this gene. Green represents upregulation and red shows downregulation.

Among the upregulated genes expressed in the media with different nitrogen sources, some were responsible for formation of the precursor nucleotide sugars that provide elements of oligosaccharide units (Table 3). For example, *pgm* encoding phosphoglucomutase is a key enzyme in sugar–nucleotide biosynthesis that catalyzes the interconversion of glucose-6-phosphate to glucose-1-phosphate, and then generates the precursor nucleotide sugars to participate in the biosynthesis of EPS in *S. thermophilus* MAU20561 (Levander and Rådström, 2001). *Gal*K encodes galactokinase in the Leloir pathway, which catalyzes the synthesis of sugar-1-phosphates.

#### 3.6.3 Analysis of eps gene clusters

The results of the expression analysis of genes in the *eps* gene cluster involved in EPS biosynthesis are shown in Table 4. Genes encoding glycosyl transferases and transporter proteins were downregulated when soy peptone was the sole nitrogen source, while the expression of *gene* 0924, *gene* 0925 involved in regulating EPS biosynthesis were upregulated. The expression of *gene* 0924, *gene* 0922, *gene* 0907, *gene* 0909, and *gene* 0910i was upregulated when tryptone was the sole nitrogen source. A total of five genes involved in phosphorylation and transposase were upregulated when casein peptone was the sole nitrogen source. When full M17 medium with a complex nitrogen source was used for the culture, the expression of *gene* 0912, *gene* 0911, and *gene* 0903 was downregulated, while the expression of *gene* 0910 encoding transposase was upregulated. Comparing the cultures at 10 h vs. 5 h, *gene* 0911, *gene* 0914, and *gene* 0919 were downregulated, indicating that the expression of these genes was higher at 5 h than at 10 h.

**Table 4:** Expression analysis results for genes involved in the biosynthesis of EPS.

**Gene ID**	**Gene names**	**1_10 h/1_5 h**		**2_10 h/2_5 h**		**3_10 h/3_5 h**		**4_10 h/4_5 h**	
		**Log**_2_ **FC**	***p*** **adjust**	**Log**_2_ **FC**	***p*** **adjust**	**Log**_2_ **FC**	***p*** **adjust**	**Log**_2_ **FC**	***p*** **adjust**
gene0903	*eps X*	−1.293 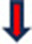	9.84E−30	−0.581 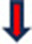	4.79E−17	−0.629 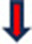	1.04E−15	−1.437 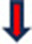	7.57E−48
gene0904	Phosphoglycerate mutase	−0.605 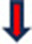	3.95E−01	−0.166 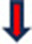	7.49E−01	0.483 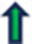	2.60E−01	0.392 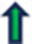	5.20E−01
gene0905	Hypothetical protein	−1.017 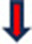	3.58E−05	−0.083 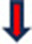	7.05E−01	0.507 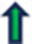	2.59E−03	−0.037 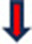	8.88E−01
gene0906	Phosphatase	−1.521 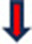	1.57E−01	−1.172 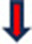	1.54E−01	−0.147 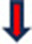	8.44E−01	0.608 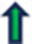	4.91E−01
gene0907	*eps O*	−0.551 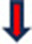	7.31E−04	0.394 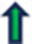	1.09E−03	0.949 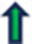	8.81E−20	0.528 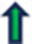	5.66E−05
gene0908	Hypothetical protein	0.000	1.00E+00	0.000	1.00E+00	0.000	1.00E+00	0.000	1.00E+00
gene0909	*Orf 14.9*	0.136 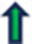	1.28E−01	0.275 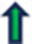	7.21E−04	0.641 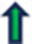	4.04E−18	0.168 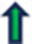	1.65E−01
gene0910	Transposase	0.740 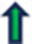	3.25E−01	0.068 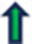	9.12E−01	0.764 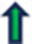	1.58E−01	1.057 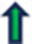	3.82E−02
gene0911	Glycosyl transferase family 1	−1.126 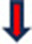	1.63E−09	−0.307 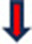	3.97E−02	−0.896 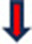	5.57E−08	−0.661 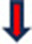	7.33E−04
gene0912	Teichoic acid transporter	−0.941 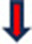	3.21E−06	−1.272 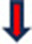	5.53E−22	−1.666 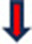	1.09E−22	−1.285 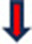	2.50E−09
gene0913	Hypothetical protein	−1.280 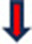	1.17E−04	−0.158 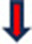	5.03E−01	−0.745 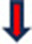	5.52E−04	−0.327 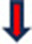	2.39E−01
gene0914	Glycosyl transferase family 2	−0.596 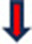	1.10E−02	−0.132 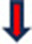	4.27E−01	−0.695 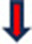	5.85E−05	−0.418 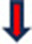	6.13E−02
gene0915	*eps F*	−1.035 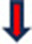	1.96E−10	−0.616 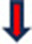	1.13E−08	−0.901 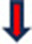	6.86E−14	−0.188 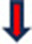	2.86E−01
gene0916	Capsular biosynthesis protein	−0.839 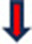	1.79E−02	−0.935 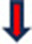	1.31E−04	−0.945 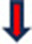	7.82E−04	−0.136 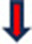	7.68E−01
gene0917	*eps H*	−0.824 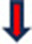	3.27E−03	−0.045 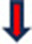	8.23E−01	−0.586 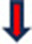	8.03E−04	−0.222 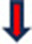	3.73E−01
gene0918	Putative capsular polysaccharide biosynthesis protein	−0.684 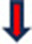	8.93E−03	−0.565 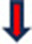	1.98E−04	−0.660 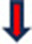	5.25E−04	0.320 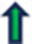	1.93E−01
gene0919	Glycosyl transferase family 1	−0.474 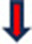	2.55E−02	−0.304 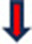	1.48E−02	−0.669 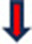	4.77E−07	0.088 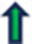	6.37E−01
gene0920	*eps 9F*	0.084 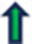	6.33E−01	−0.123 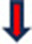	2.20E−01	−0.546 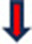	2.23E−07	0.415 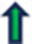	2.94E−03
gene0921	*eps E*	−0.140 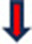	2.52E−01	−0.663 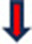	3.69E−18	−1.146 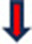	1.34E−36	−0.373 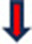	7.04E−03
gene0922	*eps D*	−0.136 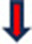	4.81E−01	0.331 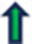	1.74E−03	−0.257 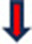	6.36E−02	0.139 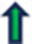	4.45E−01
gene0923	*eps C*	−0.578 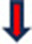	3.20E−03	0.071 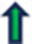	6.44E−01	−0.481 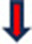	6.76E−04	0.325 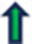	6.77E−02
gene0924	*eps B*	0.110 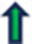	5.04E−01	0.514 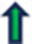	3.39E−08	−0.056 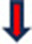	6.70E−01	0.521 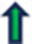	6.48E−05
gene0925	*eps A*	0.469 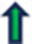	9.12E−05	−0.038 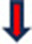	6.79E−01	−0.569 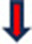	2.13E−09	0.255 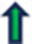	2.44E−02
gene0926	*Deo D*	0.210 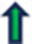	2.77E−03	−0.765 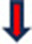	9.25E−30	−0.670 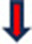	2.19E−28	0.108 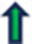	2.03E−01

1_10 h/1_5 h represents the 10 and 5 h gene expression analysis results of IMAU20561 in soy protein medium; 2_10h/2_5 h represents the 10 and 5 h gene expression analysis results of IMAU20561in tryptone medium; 3_10 h/3_5 h represents the 10 and 5 h gene expression analysis results of IMAU20561 in casein peptone medium; 4_10 h/4_5 h represents the 10 and 5 h gene expression analysis results of IMAU20561 in M17 medium; Log2FC (stress/control): the logarithm value of the difference factor of this gene between two samples with the base of 2; p adjust: test result of the significant difference between the two samples of this gene. Green represents upregulation and red shows downregulation.

### 3.7 Analysis of qRT-PCR gene expression

Twelve genes involved in sugar synthesis, glycolysis and sugar transport were investigated to determine the accuracy of the transcriptome data by qRT-PCR (Table 5). According to the results of the transcriptome data analysis, at 10 h vs. 5 h, 12 genes were upregulated, which was consistent with the RT-qPCR analysis results, indicating that the above transcriptome data is valid.

**Table 5:** Gene expression analysis of qRT-PCR.

**Group**	**Gene ID**	**Gene names**	**Log_2_FC (RNA-seq)**	**–*ΔΔ*Ct**
1_10 h_vs_1_5 h	gene0861	*malQ*	1.001	1.256
	gene1252	*pfkA*	1.177	1.530
	gene1747	*lacZ*	1.382	1.662
2_10 h_vs_2_5 h	gene0359	*glnA*	0.604	0.593
	gene1345	*nadE*	1.136	0.613
	gene1691	*manX*	0.642	0.486
3_10 h_vs_3_5 h	gene0526	*dgs*	0.603	0.624
	gene1059	*celB*	0.629	0.631
	gene1345	*nadE*	1.391	1.471
4_10 h_vs_4_5 h	gene0313	*sacA*	0.813	0.719
	gene1747	*lacZ*	0.706	1.035
	gene1289	*galE*	0.684	0.572

## 4 Discussion

We report a circular graphical map and an *eps* gene cluster in *S. thermophilus* MAU20561 isolated from a naturally fermented dairy product. The large quantity of data obtained from the transcriptomic analysis when different nitrogen sources were used allowed us to systematically investigate the mechanisms of EPS biosynthesis in *S. thermophilus* MAU20561. Alexandraki et al. (2019) noted the presence of EPS gene clusters that are present in all the *S. thermophilus* strains when investigating 23 *S. thermophilus* and the EPS clusters of different strains were compared suggesting variations in the gene content of these loci. Previously, the size of the *S. thermophilus* genome was estimated to be 1.82–1.85 Mb and about 2,000 genes involved in cell growth and metabolism were encoded (O'Sullivan and Fitzgerald, 1998). The biosynthesis of EPS is controlled by the *eps* gene cluster in *S. thermophilus* (Lavelle et al., 2022). It is generally considered that the *S. thermophilus eps* gene cluster is almost located on the chromosomal DNA and therefore the probability of losing the *eps* gene involved in the biosynthesis of EPS during the passage is low (Lavelle et al., 2022). In this study, using genomic resequencing and bioinformatics analysis, a complete *eps* gene cluster (22.3 kb), including 24 genes, was identified on the *S. thermophilus* IMAU20561 chromosomal DNA, which is responsible for the regulation of EPS biosynthesis, output, and aggregation (Figure 5).

**Figure 9:**
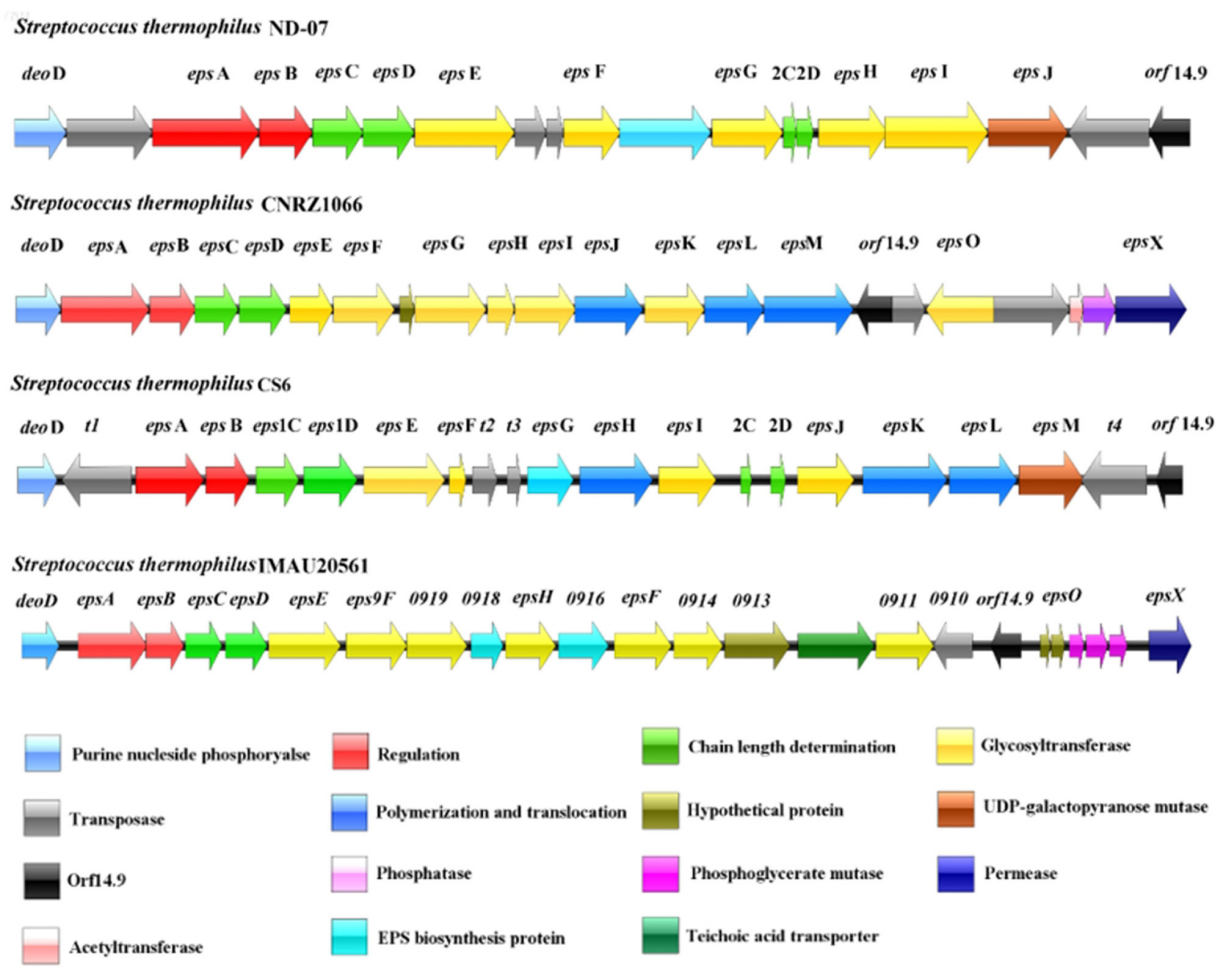
Schematic genetic organization of the eps gene cluster of *Streptococcus thermophilus* IMAU20561 compared with three other sequenced *Streptococcus. thermophilus* eps gene clusters. The predicated functions of each gene are indicated in the bottom panel in different colors.

*Streptococcus thermophilus* ND07, CNRZ1066, and CS6 were used as controls to compare the *eps* gene cluster among the experimental strains (Figure 9). The genes *eps*A, *eps*B, *eps*C, and *eps*D were highly conserved in all the *eps* gene clusters and are responsible for EPS regulation, chain length and polymerization. These genes were found in both *S. thermophilus* IMAU20561 and the other three *S. thermophilus eps* gene clusters and they were also found to appear in the same order. Glycosyltransferases play a key role in the biosynthesis of the EPS repeating unit, and the type and numbers of these enzymes in the *eps* gene cluster determines the diversity of EPS structure (Dan et al., 2009; Wu et al., 2014). A diversity of glycosyltransferases can transfer the sugar residues of nucleotide sugars to an acceptor, thus suggesting that the biosynthesis of EPS probably requires a lot of glycosyltransferase genes (Breton et al., 2006). Seven putative glycosyltransferase genes that had been identified previously were similar in *S. thermophilus* IMAU20561 as well. The number and type of genes regulating polymerization and translocation varied among the strains. Among them, *eps*O was oriented in the opposite direction to the *eps* gene cluster. The preliminary chemical evaluation of the EPS of *S. thermophilus* IMAU20561 indicated that the monosaccharaides mannose, glucose, and galactose were present in the EPS (Liu et al., 2022).

The annotated DEGs responsible for the production of EPS under different nitrogen sources in *S. thermophilus* IMAU20561 were investigated using GO and KEGG pathway analysis. From the results of the enrichment analysis of DEGs with GO function, the top 10 significant GO terms are visualized in Supplementary Table S1 based on FDR values. From GO enrichment analysis, it is found that DEGs between different nitrogen sources were mainly enriched in amino acid biosynthesis and metabolism; biosynthesis and metabolism of ribonucleotides; IMP biosynthesis and metabolism; and phosphorus metabolism processes. KEGG enrichment analysis also showed significant enrichment of pathways involved in amino acid metabolism under different nitrogen sources. Furthermore, qPCR technology can accurately describe the gene expression level and has become a common method for the detection of gene expression. In this study, 12 genes related to sugar transport, sugar synthesis and glycolysis were selected and their expressions were analyzed by RT-qPCR. The up-regulation trend of 12 genes was consistent with the up-regulation trend of differential genes analyzed in transcriptome data. The qPCR analytic results confirmed the accuracy of RNA-seq data.

EPSs from LAB have a broad application potential in agri-food as a substitute for food-grade bioamendments and additives. In the dairy industry, EPSs are used as biothickeners due to their stabilizing, emulsifying or gel properties. However, the commercial output of EPS is relatively low and further research is needed to improve the yield of EPS and promote its development in the field of food and agri-culture. Currently, the structure and biological activity of EPS derived from LAB and the relationship between EPS genes, phenotype, structure, and function have been receiving increased research attention. This provides a theoretical basis for the practical application in future and also information that could improve the yield and structure of EPS through a genetic means.

In summary, we report the complete genome sequence of *S. thermophilus* MAU20561, which contains 1,716,258 bp encoding 1,914 coding sequences (CDSs). Among them, a 22.3-kb *eps* gene cluster that also includes 24 genes was identified. The results of GO and KEGG functional annotation showed that upregulated DEGs were mainly involved in amino acids, ribonucleotide, glycolysis, phosphotransferase system, fructose, and mannose metabolism and accounted for improving the production of EPS by *S. thermophilus* MAU20561. This work provides new insights into genetic characteristics of *S. thermophilus*, biosynthetic pathways for the production of EPS and a theoretical basis for screening dairy starter cultures.

## Data availability statement

The original contributions presented in the study are included in the article/Supplementary material, further inquiries can be directed to the corresponding author.

## Author contributions

YW: Methodology, Resources, Supervision, Writing—original draft, Writing—review & editing, Investigation, Software. QP: Conceptualization, Investigation, Methodology, Visualization, Writing—original draft, Formal analysis, Validation. YL: Data curation, Methodology, Validation, Writing—original draft. NW: Data curation, Methodology, Writing—original draft. YH: Methodology, Software, Writing—original draft. XC: Formal analysis, Methodology, Writing—original draft. TD: Conceptualization, Data curation, Funding acquisition, Investigation, Methodology, Project administration, Resources, Writing—review & editing.
