# Supplementary material for: Genomic and transcriptomic analysis of genes involved in exopolysaccharide biosynthesis by Streptococcus thermophilus IMAU20561 grown on different sources of nitrogen
Source: Front Microbiol. 2024 Jan 29;14:1328824. doi: 10.3389/fmicb.2023.1328824 (PMC10859522; doi:10.3389/fmicb.2023.1328824)
Supplement: Supplementary file 1 [file Table_1.DOCX]

**Table S1.** GO enrichment analysis of DEGs

| Sample names | GOID | Representative gene |
| --- | --- | --- |
|  |  |  |
| 1_5 h_vs_1_10 h | histidine biosynthetic process，imidazole-containing compound metabolic process，histidine metabolic process，cellular biogenic amine metabolic process, indole-containing compound metabolic process | *gene*0877，*gene*1818，*gene*0875 |
| 2_5 h_vs_2_10 h | peptide metabolic process，translation，structural molecule activity，structural constituent of ribosome，intracellular ribonucleoprotein complex | *gene*0873，*gene*1382，*gene*1018 |
| 3_5 h_vs_3_10 h | protein metabolic process，cytoplasmic part，rRNA binding，cellular protein metabolic process，peptide biosynthetic process | *gene*1109，*gene*1108，*gene*1189， |
| 4_5 h_vs_4_10 h | IMP metabolic process，IMP biosynthetic process，'de novo' IMP biosynthetic process，nucleoside monophosphate biosynthetic process，carbohydrate derivative catabolic process | *gene*0880，*gene*1106，*gene*0216， |
| 1_5 h_vs_2_5 h | transferase activity, transferring alkyl or aryl (other than methyl) groups，ion channel activity，carboxypeptidase activity，gated channel activity，substrate-specific channel activity | *gene*0877，*gene*1181，*gene*0878， |
| 1_5 h_vs_3_5 h | IMP metabolic process，IMP biosynthetic process，'de novo' IMP biosynthetic process，S-adenosylmethionine-dependentmethyltransferase activity，rRNA methyltransferase activity | *gene*1416，*gene*1695，*gene*1330， |
| 1_5 h_vs_4_5 h | de novo' IMP biosynthetic process，ribonucleoside monophosphate metabolic process，nucleoside monophosphate metabolic process，IMP metabolic process，IMP biosynthetic process | *gene*1632，*gene*1024，*gene*1032， |
| 1_10 h_vs_2_10 h | de novo' IMP biosynthetic process，imidazole-containing compound metabolic process，histidine metabolic process，IMP metabolic process，IMP biosynthetic process | *gene*0518，*gene*0216，*gene*1746 |
| 1_10 h_vs_3_10 h | de novo' IMP biosynthetic process，IMP metabolic process，IMP biosynthetic process，phosphate-containing compound metabolic process，nucleoside phosphate biosynthetic process | *gene*1373，*gene*1107，*gene*1106， |
| 1_10 h_vs_4_10 h | de novo' UMP biosynthetic process，intrinsic component of membrane，integral component of membrane，pyrimidine ribonucleoside monophosphate metabolic process，UMP metabolic process | *gene*1024，*gene*1822，*gene*1292， |
| 2_5 h_vs_3_5 h | phosphoenolpyruvate-dependent sugar phosphotransferase system，protein-N(PI)-phosphohistidine-sugar phosphotransferase activity，carbohydrate transport，membrane part，carbohydrate transmembrane transporter activity | *gene*0877，*gene*0872，*gene*0870， |
| 2_5 h_vs_4_5 h | carbon-oxygen lyase activity，lyase activity，hydro-lyase activity，leucine metabolic process，leucine biosynthetic process，solute:cation symporter activity | *gene*1747，*gene*0457，*gene*0898 |
| 2_10 h_vs_3_10 h | metal ion transport，cysteine metabolic process，cation transport，cysteine-type peptidase activity，sulfur amino acid metabolic process | *gene*0870，*gene*0878，*gene*1923， |
| 2_10 h_vs_4_10 h | histidine biosynthetic process，imidazole-containing compound metabolic process，histidine metabolic process，IMP metabolic process，IMP biosynthetic process | *gene*1921，*gene*0870，*gene*1272， |
| 3_5 h_vs_4_5 h | de novo' UMP biosynthetic process，ribonucleoside monophosphate biosynthetic process，nucleoside monophosphate biosynthetic process，pyrimidine ribonucleoside monophosphate biosynthetic process，pyrimidine ribonucleoside monophosphate metabolic process | *gene*1805，*gene*1806，*gene*1807， |
| 3_10 h_vs_4_10 h | IMP metabolic process，IMP biosynthetic process，'de novo' IMP biosynthetic process，purine ribonucleoside monophosphate metabolic process，purine nucleoside monophosphate metabolic process | *gene*0996，*gene*0518，*gene*0878， |
