# Supplementary material for: Genomic and transcriptomic analysis of genes involved in exopolysaccharide biosynthesis by Streptococcus thermophilus IMAU20561 grown on different sources of nitrogen
Source: Front Microbiol. 2024 Jan 29;14:1328824. doi: 10.3389/fmicb.2023.1328824 (PMC10859522; doi:10.3389/fmicb.2023.1328824)
Supplement: Supplementary file 2 [file Table_2.DOCX]

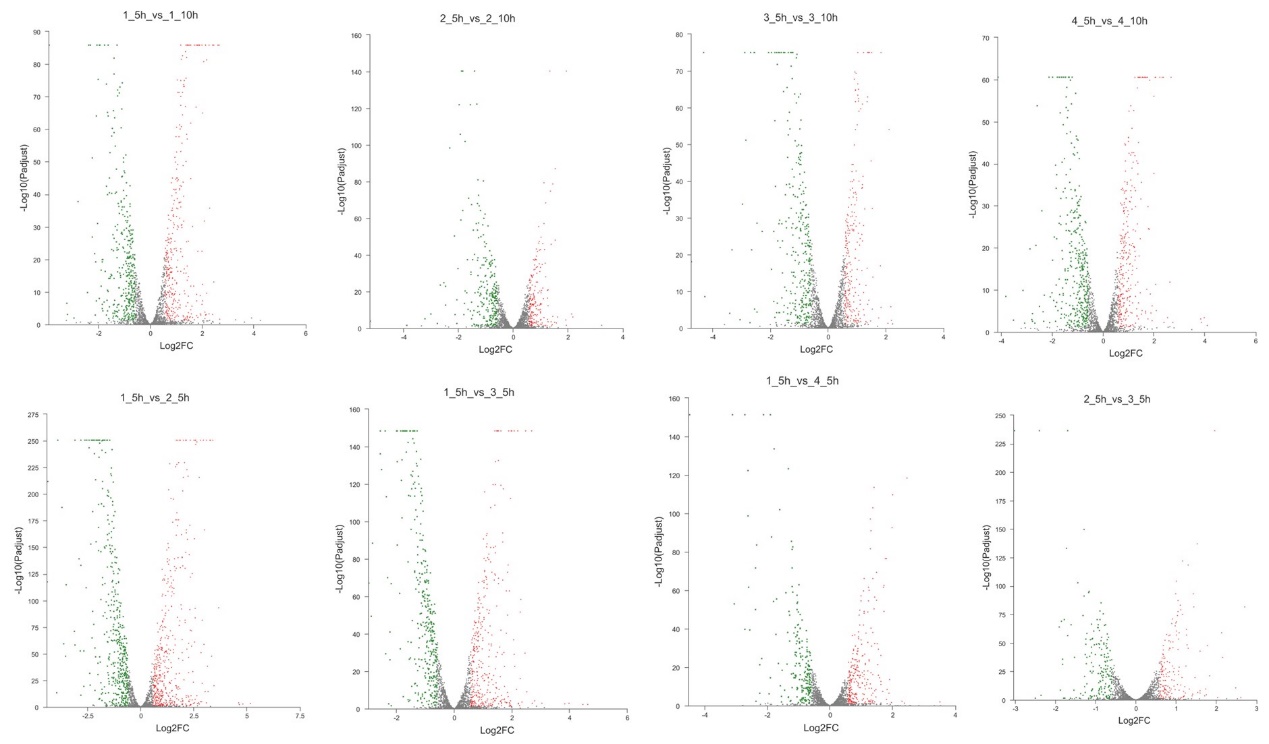


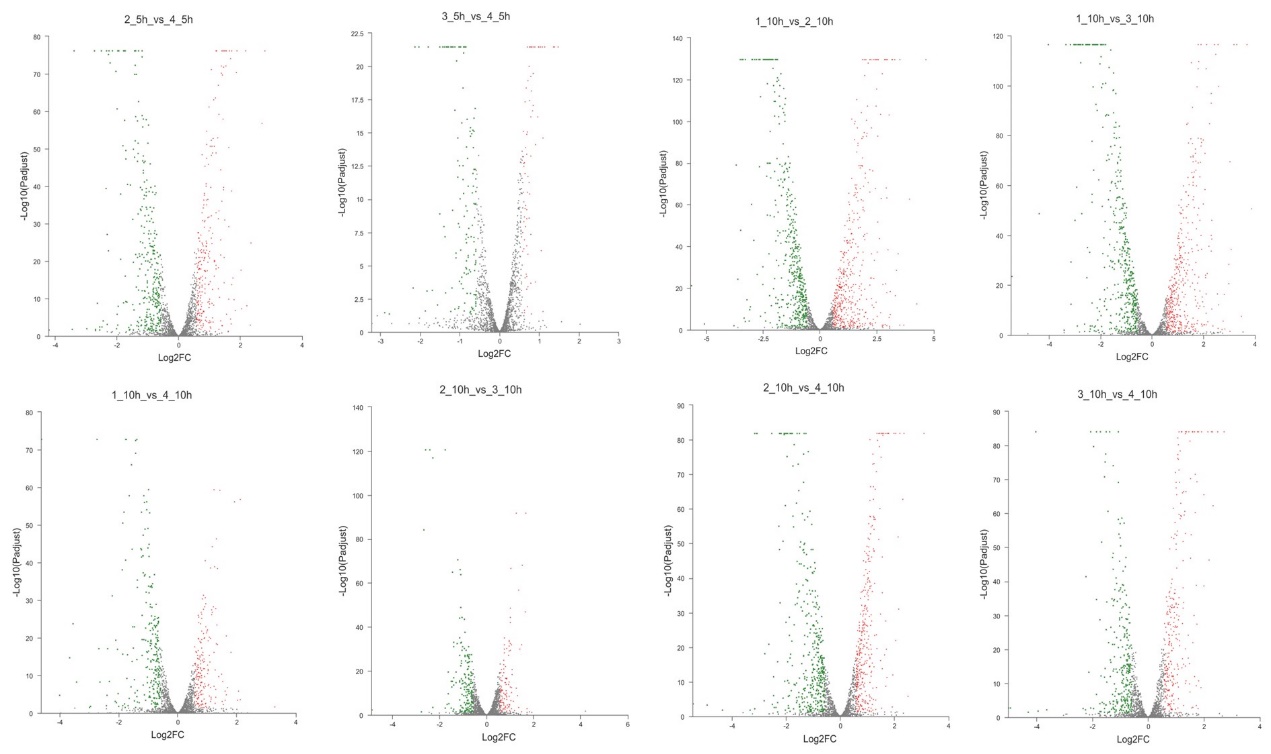


**Figure S1.** Volcano map of *S. thermophilus* MAU20561 at 5 h and 10 h. The abscissa

represent differences in gene expression between the two samples. The value is

logarithmically processed and it is the P value that represents the statistical significance

of differences in gene expression. The red dots indicate genes that are significantly up--regulated, the green dots indicate genes that are significantly down--regulated, and the grey dots indicate genes that showed no significant change in regulation. 1-3 = M17 medium supplied soy peptone, tryptone, or casein peptone; 4 = labeled M17 medium.
