# Supplementary material for: Genomic and transcriptomic analysis of genes involved in exopolysaccharide biosynthesis by Streptococcus thermophilus IMAU20561 grown on different sources of nitrogen
Source: Front Microbiol. 2024 Jan 29;14:1328824. doi: 10.3389/fmicb.2023.1328824 (PMC10859522; doi:10.3389/fmicb.2023.1328824)
Supplement: Supplementary file 3 [file Table_3.DOCX]

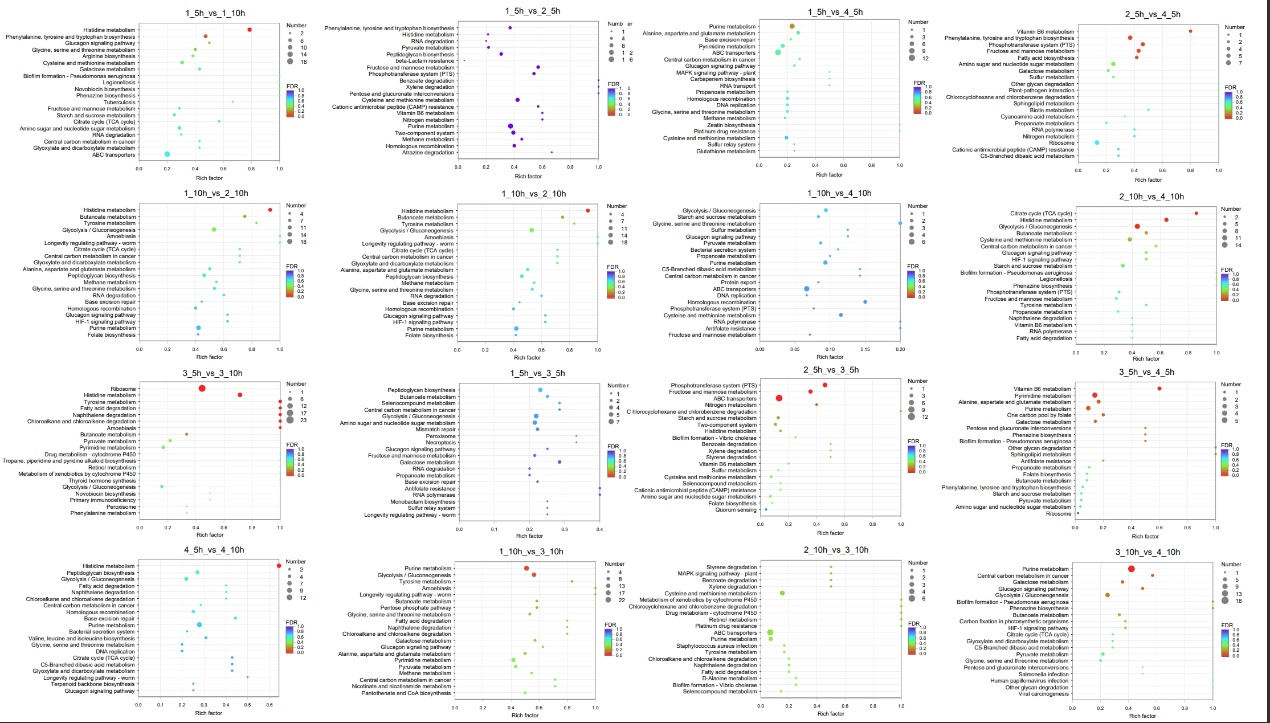


**Figure S2.** KEGG pathway enrichment scatter plot. The Y-axis shows the KEGG pathway, the X-axis shows the rich factor for each pathway. The dot size indicates the number of DEGs in the pathway, and the dot colour indicates FDR values.
